# Supplementary material for: Highly Efficient NO2 Sensors Based on Al-ZnOHF under UV Assistance
Source: Materials (Basel). 2023 May 7;16(9):3577. doi: 10.3390/ma16093577 (PMC10180258; doi:10.3390/ma16093577)
Supplement: Supplementary file 1 [file materials-16-03577-s001.zip › materials-2358539-supplementary.pdf]

Supporting information for:

Highly Efficient NO_2 Sensors Based on Al-ZnOHF under UV Assistance

Xingyu Yao [1], Rutao Wang [1], Lili Wu [1,*], Haixiang Song [2], Jinbo Zhao [3], Fei Liu [1], Kaili Fu [1], Zhou Wang [1], Fenglong Wang [1] and Jiurong Liu [1]

[1] Key Laboratory for Liquid-Solid Structural Evolution and Processing of Materials, Ministry of Education and School of Materials Science and Engineering, Shandong University, Jinan 250061, China
[2] Henan Joint International Research Laboratory of Nanocomposite Sensing Materials, School of Chemical and Environmental Engineering, Anyang Institute of Technology, Anyang 455000, China.
[3] School of Materials Science and Engineering, Qilu University of Technology (Shandong Academy of Sciences), Jinan 250353, China
* Correspondence: wulili@sdu.edu.cn

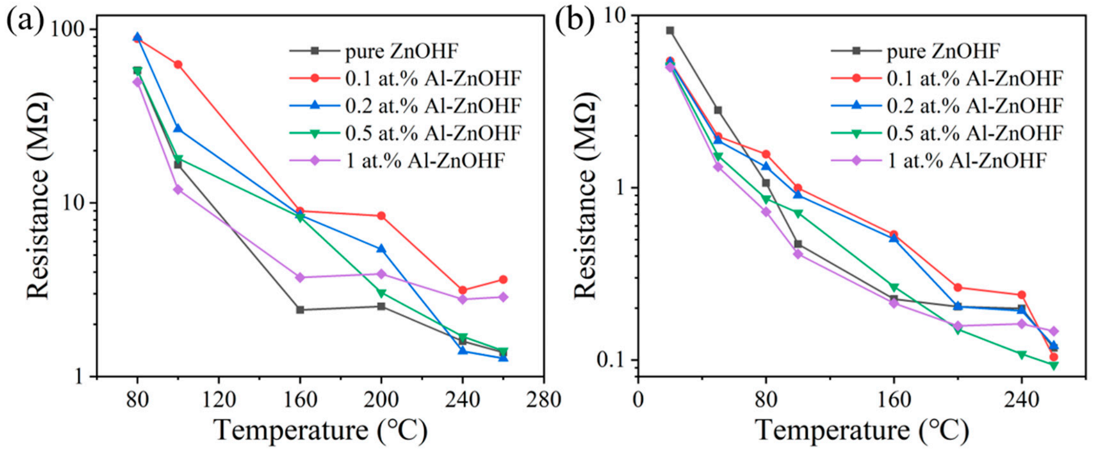

Figure S1. the resistance of samples at different temperature (a) without, (b) with UV radiation

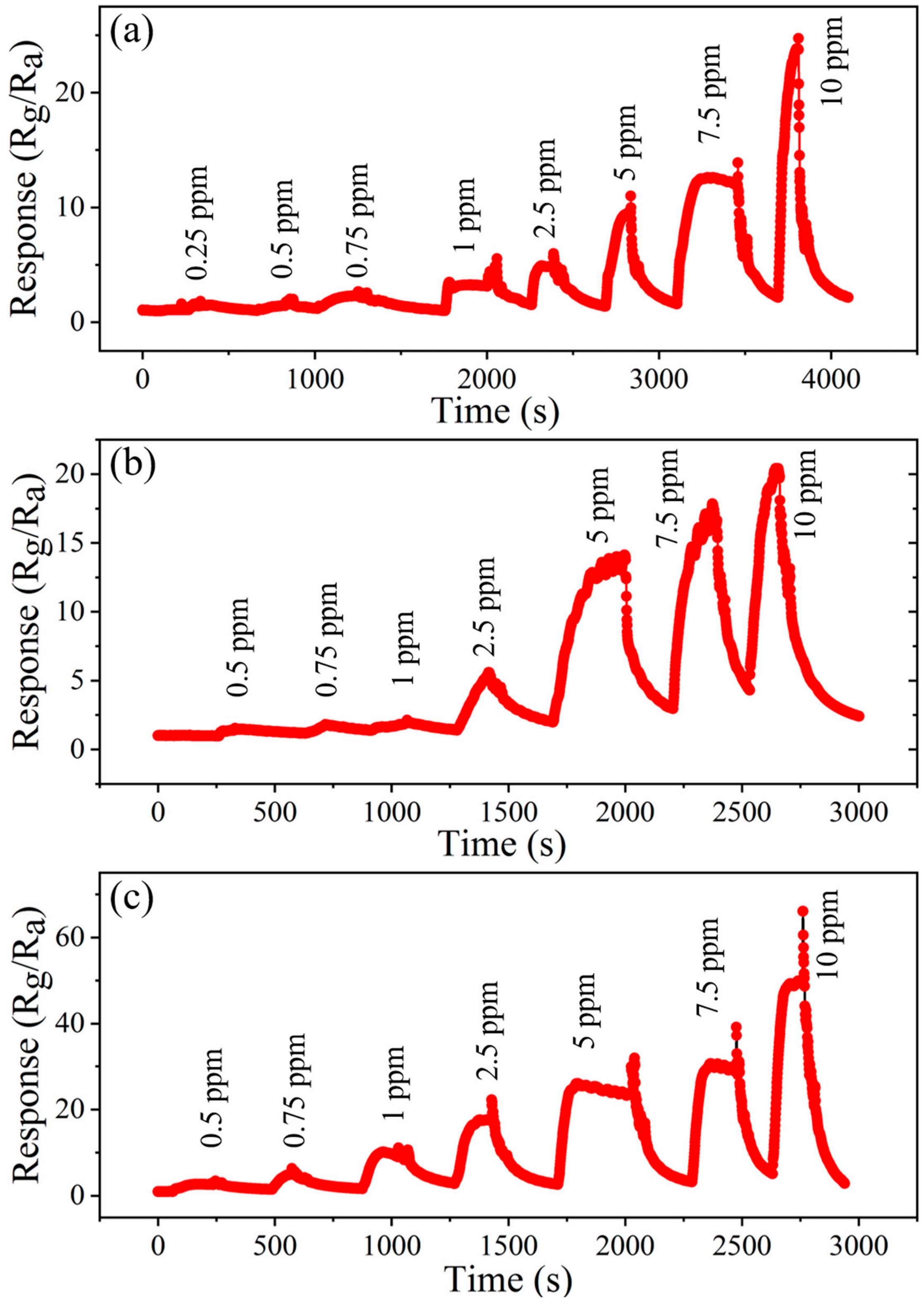

Figure S2. the dynamic response curve of (a) pure ZnOHF with UV assistance, 100 ℃, (b)pure ZnOHF in dark, 160 ℃ and (c) 0.5 at.% Al-ZnOHF in dark, 160 ℃
